# Increased Intake of Vegetables and Fruits Improves Cognitive Function among Chinese Oldest Old: 10-Year Follow-Up Study

**DOI:** 10.3390/nu15092147

**Published:** 2023-04-29

**Authors:** Afei Qin, Meiqi Wang, Lingzhong Xu

**Affiliations:** 1 Centre for Health Management and Policy Research, School of Public Health, Cheeloo College of Medicine, Shandong University, Jinan 250012, China; qaf22377@163.com (A.Q.); wmq20000220@163.com (M.W.); 2 National Health Commission (NHC) Key Laboratory of Health Economics and Policy Research, Shandong University, Jinan 250012, China; 3 Center for Health Economics Experiment and Public Policy Research, Shandong University, Jinan 250012, China

**Keywords:** vegetables and fruits, intake pattern, generalized-estimating equations, longitudinal study, mild cognitive impairment, Chinese oldest old

## Abstract

Background: In view of the rapidly accelerating aging process in China, this study looked at the associations between vegetables and fruits intake pattens and cognitive function among the oldest old in China using the genetic sub study from the Chinese Longitudinal Healthy Longevity Survey (CLHLS). Methods: This study screened respondents who participated in all four surveys of longitudinal data from the CLHLS, and a total of 2454 participants were ultimately included. The relationships of cognitive function with vegetables and fruits intake patterns were examined using Generalized-estimating equations. Results: The prevalence range of mild cognitive impairment (MCI) was 14.3% to 16.9% at T1 to T3 and 32.7% at T4. There was a significant increase in the prevalence of MCI from T1 to T4 (β = 0.054; 95% CI, 0.037 to 0.070; *p <* 0.001; adjusted). The V+/F+ pattern significantly improved cognitive function in Chinese older adults compared with the V−/F− pattern (OR, 1.026; 95% CI, 1.001–1.053; *p* < 0.05). Conclusion: Older adults who frequently consume both fruits and vegetables experience a reduction in MCI risk relative to those consuming these food groups infrequently—emphasizing the critical importance of the regular intake of both fruits and vegetables in maintaining cognitive function.

## 1. Introduction

Global population aging is an actuality. In the year 2019, there were 703 million people worldwide who were 65 or older [1]. In 2050, this figure is anticipated to increase to 1.5 billion. China, which continues to have the largest population in the world as of today, is also rapidly aging. According to the most recent 7th national census, there will be 190.6 million Chinese people 65 and over in 2020, or 13.5% of the whole Chinese population [2]. A common symptom linked to aging is mild cognitive impairment (MCI) [3]. According to estimates, in those older than 65, the prevalence of MCI ranges from 3% to 22% [4], but it was 9.7% to 23.3% in China [5,6], and more than half of MCI patients suffered from Alzheimer’s disease (AD) [7]. Age is the biggest risk factor among many that raise the likelihood of having MCI. MCI is now a public health problem since it is an age-related disorder. There is currently no effective medication or therapy for AD prevention. To stop MCI patients from transitioning into AD, it is crucial to discover potential protective factors. The lifestyle, longevity, urbanization, and environmental changes that have occurred in China over the past several decades are expected to have an impact on the exposure to risk factors for Alzheimer’s disease and, therefore, the prevalence of dementia. Systematic risk factors for dementia or MCI, both changeable variables and immutable risk factors, have been evaluated in prior research [8]. In addition to these other risk factors, dietary health may also be a significant risk factor for MCI. Diet has an impact on the diversity and health of the gut microbiota [9]. Eating habits are directly associated with AD because changes in gut microbial composition may have an impact on the brain’s physiological, behavioral, and cognitive functions [10,11,12]. According to a prospective study on older Japanese participants, increasing consumption of fermented dairy products may help reduce the prevalence of dementia, including AD [13]. According to reports, AD is less prevalent among Hainan Tibetans in Qinghai than it is among populations in other nations. This may be because to the Tibetan population’s unique dietary practices and other customs and religious beliefs [14].

The most well-established connection between dietary patterns and MCI is that between the consumption of fruits and vegetables and MCI. Consuming fruits, vegetables, and juices regularly may improve cognition in healthy older persons, according to research [15]. Anthocyanin-rich fruits, for example, have been reported to reduce TNF-α concentration in older adults with MCI [16]. Moreover, adherence to the Mediterranean-DASH Intervention for Neurodegenerative Delay (MIND) eating pattern, which emphasizes berries and leafy greens, has also shown promise in improving cognitive outcomes [17]. In the Malaysian population, the promotion of “tropical fruit-oats” intake serves as a preventive measure against dementia in the older adults [18]. Cohort studies indicate that the regular consumption of fruits and certain vegetables may mitigate age-related cognitive losses among the middle aged [19]. The relationship between consuming fruits and vegetables and cognition in many Western nations has been the subject of increasing research. For instance, research from 11 European nations demonstrated that eating fruits and vegetables often was linked to better cognitive and mental health [20]. According to Swedish research, eating more fruits and vegetables may lower the risk of getting dementia. This relationship has been confirmed in investigations of American, French, and Spanish speakers [21,22,23]. A significant plant consumption has also been linked in several studies to the maintenance of cognition [24,25,26], with the benefit of combined fruit and vegetable intake being more prominent. Despite the paucity of data from Asian cultures, several studies have shown links between fruit and vegetable consumption and cognitive decline [27]. The cognitive function of elderly Japanese individuals may be enhanced by a diet heavy in fruits and vegetables [28]. Poor cognitive function was linked in cross-sectional analyses to reduced overall vegetable consumption in Korean women [29].

Numerous studies have revealed a link between regular fruit and vegetable consumption and a lower risk of illness [30,31,32]. Even the higher the intake of fruits and vegetables, the lower the mortality rate [33]. Fruits and vegetables may synergistically act to safeguard cognitive function. Research has demonstrated that the combined effects of individual phytochemicals exceed the sum of their respective biological contributions [34]. Hence, it is plausible that the joint intake of both fruits and vegetables may offer greater neuroprotection than consuming either one alone. A substantial body of research underscores the benefits of regular fruit and vegetable consumption in enhancing cognitive outcomes among older adults worldwide, including Asia and China. Although some studies of older Chinese populations have identified similar relationships, there remains a paucity of national or cohort data for China as a whole. Additionally, investigations on the combined effects of fruit and vegetable intake in China are comparatively scarce.

For the reasons mentioned above, we used the genetic sub study from the Chinese Longitudinal Healthy Longevity Survey (CLHLS), a large-scale community-based study, to examine the relationships between the frequency and patterns of vegetable and fruit consumption and cognitive function among the oldest old in China. In addition to examining the connection between eating fruit and vegetables and cognitive function, our research also examined the effect of the frequency of vegetable and fruit intake as two separate factors on MCI and compared the differences between the two results to examine synergies. The effects of other potential confounding factors on cognitive function, such as lifestyle conditions and depression, were also tested. In consideration to the rapidly accelerating aging process in China, this research is significant for encouraging seniors to adopt healthy eating habits and enhance their cognitive abilities, as well as minimizing the future burden of MCI and AD on families and society.

## 2. Materials and Methods

### 2.1. Study Design and Sample

The China Longitudinal Healthy Longevity Survey (CLHLS; The 2008–2018 tracking data set 2008; 2008–2009, 2011–2012, 2014 and 2017–2018) provided the longitudinal data for this investigation over four waves. In order to fill information and knowledge gaps for research studies and policy analyses on healthy aging, the CLHLS has been carried out in China from 1998. The initial survey was performed in 1998, and further surveys were carried out in 2000, 2002, 2005, 2008–2009, 2011–2012, 2014, and 2017–2018. Established by researchers and investigators from Duke University, Peking University, and other institutions, CLHLS is a continuing open cohort that has been part of a worldwide collaboration. The CLHLS dataset is the first longitudinal dataset to examine the health and longevity of Chinese older people, concentrating on the oldest old in a developing country. The CLHLS has been collecting considerable longitudinal home interview data on a far larger sample of oldest old aged 80 and older than has previously been investigated. Health status, disability, mortality, and survival, as well as demographics, household, socioeconomic factors, income levels, care requirements, costs of older persons, and behavioral risk factors associated with mortality and healthy aging, are all significant metrics included. The CLHLS is a national survey of China that was carried out in randomly chosen counties and cities in 22 of the country’s 30 provinces, covering 85% of the country’s population. More detailed information about this dataset was described elsewhere [35,36]. The project was approved by the Biomedical Ethics Committee of Peking University, China (IRB00001052-13074). In the baseline and follow-up questionnaires, all participants completed written consent forms or provided written consent through their legal representative.

The present data were obtained at 4 time points: T1, data collected in 2008–2009, a sample of 16,954 elderly people aged 65 years and older; T2, data collected in 2011–2012, a sample of 9765 elderly people aged 65 years and older; T3, data collected in 2014, a sample of 7192 elderly people aged 65 years and older; and T4, data collected in 2017–2018, a sample of 15,874 elderly people aged 65 years and older. This study screened respondents who participated in all four surveys and a total of 2454 participants were ultimately included.

### 2.2. Measures

#### 2.2.1. Assessment of Cognitive Function

The Chinese version of the Mini-mental State Examination (MMSE), which comprises 24 indicators that indicate the orientation, responsiveness, attention and calculation, memory, language comprehension, and coordination of the elderly, was used to evaluate the cognitive function of CLHLS participants. The MMSE has a value range of 0 to 30, and greater MMSE scores correspond to higher levels of cognitive function. Numerous earlier studies [37,38] have shown the reliability and validity of this Chinese MMSE. According to earlier research, an MMSE score of less than 24 was considered to be indicative of MCI (MCI is defined as MMSE = 0, otherwise MMSE = 1) [39,40,41].

#### 2.2.2. Assessment of Vegetable and Fruit Intake Frequency

Vegetable and fruit intake frequencies were assessed by asking “do you often eat fresh fruits/vegetables?”, and the options were set as “eat every day/almost every day”, “eat often”, “eat sometime”, and “eat little or never”. These two individual problems associated with measuring vegetable and fruit intake have been validated [42]. Several studies have relied on these two individual questions to ascertain the frequency of fruit and vegetable intake [43,44,45], while other investigations have employed them to classify the frequency of consumption into discrete categories [46]. In order to classify vegetable and fruit intake patterns and avoid overclassification, daily or often and sometimes or never intake categories were employed to reflect high and low consumption of vegetables and fruit, which was strongly advised by several dietary guidelines [47]. Therefore, the following four intake patterns were defined: V+/F+: daily or often intake of both vegetables and fruit; V+/F−: daily or often intake of vegetables but sometimes or never intake of fruits; V−/F+: sometimes or never intake of vegetables but daily or often intake of fruits; and V−/F−: sometimes or never intake of either vegetables or fruit.

In addition to the aforementioned classifications, this study performed a separate analysis of fruit and vegetable intake frequencies as two independent variables in order to enhance result clarity. The frequency categories were assigned quantitative values: “eat every day/almost every day” and “eat often” were classified as “1”, representing high frequency intake, while “sometimes” and “rarely or never” were classified as “0,” indicating low frequency intake. Notably, these two individual questions captured the frequency rather than the quantity of fruit and vegetable intake; therefore, all results and explanations pertained solely to frequency metrics.

#### 2.2.3. Assessment of Depression

The PhenX (consensus measurements for Phenotypes and exposures) Toolkit was used by the CLHLS to quantify depression (PhenX code: 120500). PhenX is a brief 2-item self-report screening tool that asks participants to indicate whether they have experienced depression symptoms lasting at least two weeks in the last 12 months (1 = Yes), whether they have not (2 = No), or whether they are unable to respond (8 = not able to answer). If at least one response to the questions was 1 = Yes, the participant was deemed depressed.

The reliability of PhenX was evaluated using the Spearman–Brown coefficient rather than Cronbach’s alpha because it only contained two items [48]. Phen X demonstrated strong validity and reliability in other studies [49,50]. The Spearman–Brown coefficient in this study was 0.738, demonstrating the high internal consistency and reliability of the depression measurement tool. This variable was only accessible in T2 and T3 databases due to the database scale.

#### 2.2.4. Assessment of Psychological Well-Being (PWB) 

Seven items encompassing both positive (sense of personal control, conscientiousness, and good sentiments about aging) and negative effects (optimism, loneliness, anxiety, and loss of self-worth) with a five-point Likert scale were used to evaluate PWB. There were five response levels: always, frequently, occasionally, rarely, and never. Respondents were instructed to select one of five choices. Positive PWB was indicated by a higher score since we have reversed the coding of negative emotions [51]. The reliability of these seven measurement questions has been demonstrated [52,53]. The index’s internal consistency (Cronbach’s alpha) was 0.650. Due to the database scale, this variable is only available in T1, T2, and T3 databases.

#### 2.2.5. Individual-Level Covariates

Following the previous studies, we considered potential confounders, including gender, age, education background (years of schooling), ethnicity (Han and others), residence (rural and urban), living condition (live with family, live alone, live in a nursing institution), self-assessed economic level (good, fair, poor), annual household income (Q1 = less than 25,000, Q2 = 25,000–49,999, Q3 = 50,000–74,999, Q4 = more than 75,000; measured in Renminbi (RMB), the Chinese currency), marital status (currently married and living with spouse, currently married and not living with spouse, and unmarried or other), timely treatment (illness can be treated promptly (yes) and illness cannot be treated promptly (no)), and childhood starvation (Starving (yes) and not starving (no)).

### 2.3. Statistical Analysis

Generalized-estimating equations (GEE) with an unstructured covariance matrix and a robust estimator were utilized in the statistical studies, which were carried out using IBM Corp.’s SPSS software version 27. GEE is a regression model that analyzes correlation data by combining repeated measurement data and the quasi-likelihood estimation approach to determine generalized linear model parameters. We utilized gamma and logarithmic join types in this study. The method considers the interdependence of the observation periods and incorporates all information that is readily available for each participant, making it less vulnerable to the effects of missing data. We did not add random effects to the model since we were only interested in the fixed effects of the listed components as anticipated. In the present study’s analysis, the score of MMSE was examined as the dependent variable.

First, in Model 1, we performed an unadjusted analysis of the association of all the independent variables measured at T1 (including the queue), with MCI measured at T1–T5 and then the adjusted analysis. Secondly, given that only T2–T3 data included measurements of depression, all independent variables in Model 2 (including subjective well-being and depression) came from T2 and dependent variables from T3. Then, we analyzed the frequency of vegetable and fruit intake as two independent variables; all the independent variables were measured from T1, and the dependent variables were measured from T2–T4. The relationships of cognitive function with all independent variables (including all covariates) in the two models were examined using GEE. *p* < 0.05 was used to define reported *p* values as statistically significant.

To control the false discovery rate (FDR), two-sided *p* values were corrected according to the Benjamini/Hochberg (B/H) methodology [54] to account for multiple testing. If the B/H-adjusted *p* value of an association was lower than 0.05, indicating a false discovery rate (FDR) of 5%, it was considered to be statistically significant. 

Furthermore, a normality assessment was performed on the dependent variable MCI. The Kolmogorov–Smirnoff test demonstrated a significance level of less than 0.0001 and a skewness value of −2.351 (Standard error = 0.029), and a kurtosis value of 5.741 (Standard error = 0.057) was obtained. These findings suggested that the dependent variable MCI did not conform to a normal distribution. However, utilizing a connection function, the Generalized estimating equations (GEE) method was employed to fit the dependent variables of binomial, Poisson, and Gamma distributions into corresponding statistical models, thus resolving the issue of non-independence of repeated measurement data and attaining precise parameters for dependent variables of varying distribution types. Moreover, we performed a multicollinearity assessment on all independent variables, and the findings revealed that the tolerance values of each independent variable ranged between 0.6 and 1. Likewise, the Variance Inflation Factor (VIF) values of each independent variable ranged between 1 and 1.7. Based on these outcomes, it can be inferred that no collinearity existed among the independent variables.

## 3. Results

Of the 2454 older adults who participated in the four-period survey, the mean (SD) age was 75.46 (8.21) years; 1307 (53.3%) were women; 2293 (93.65) were Han Chinese. Table 1 shows a description of the variables among the respondents.

### 3.1. Prevalence of MCI among Chinese Older Adults

Table 1 also shows the prevalence of MCI for the various time periods. The prevalence was 14.8% (362 of 2454) at T1 (70.4% (255 of 362) for women vs. 29.6% (107 of 362) for men; *p* = 0.008), 14.3% (351 of 2454) at T2 (69.5% (244 of 351) for women vs. 30.5% (107 of 351) for men; *p* = 0.076, no significant difference), 16.9% (415 of 2454) at T3 (73.0% (303 of 415) for women vs. 27.0% (112 of 415) for men; *p* < 0.001), and 32.7% (803 of 2454) at T4 (67.6% (543 of 803) for women vs. 32.4% (260 of 803) for men; *p* < 0.001). From T1 to T4, there was a discernible rise in the prevalence of MCI (β = 0.114; 95% CI, 0.101 to 0.127; *p* < 0.001; unadjusted and β = 0.054; 95% CI, 0.037 to 0.070; *p* < 0.001; adjusted).

### 3.2. Factors Associated with MCI among Chinese Older Adults

The unadjusted and adjusted factors associated with MCI among Chinese older adults in model 1 and adjusted factors associated with MCI among Chinese older adults in model 2 are shown in Table 2. In the adjusted model 1, the V+/F+ patterns significantly improved cognitive function in older adults compared with the V−/F− pattern (OR, 1.026; 95% CI, 1.001–1.053; *p <* 0.05). In addition, risk factors for cognitive function in older Chinese include female (OR, 0.962; 95% CI, 0.951–0.974; *p <* 0.001) and older age (OR, 0.992; 95% CI, 0.991–0.993; *p <* 0.001), and protective factors for cognitive function in older Chinese include longer years of schooling (OR, 1.005; 95% CI, 1.003–1.006; *p <* 0.001) and not starving in childhood (OR, 1.019; 95% CI, 1.008–1.030; *p <* 0.01). After adjustment, the PWB was significantly positively associated with cognitive function (OR, 1.003; 95% CI, 1.001–1.005; *p <* 0.01), and depression was not statistically significant in association with cognitive function (OR, 0.985; 95% CI, 0.967–1.003; *p >* 0.05), which were presented in the results of Model 2.

### 3.3. The Relationship between the Frequency of Fruit and Vegetable Consumption and MCI among Chinese Older Adults

In order to explore the effects of vegetable and fruit intake frequency on the MCI in the older adults, respectively, in model 3, fruit intake frequency and vegetable intake frequency were used as two independent variables. The results in Table 3 show that frequent fruit eating (OR, 0.989; 95% CI, 0.972–1.006; *p* > 0.05) and frequent vegetable eating (OR, 1.006; 95% CI, 0.994–1.018; *p* > 0.05) have no significant positive or negative effects on cognitive function improvement in the older adults compared with infrequent consumption.

## 4. Discussion

In this cohort research, older Chinese older adults had greater rates of MCI, particularly at T4, when the incidence of MCI reached 32.7%. The V+/F+ patterns of vegetables and fruits can greatly boost cognitive function and reduce the incidence of MCI in the older adults in China. Higher PWB scores imply greater cognitive performance, and PWB can also considerably enhance cognitive function. Depression is not related to cognitive function. Additionally, as older adults age, the possibility of them suffering from MCI increases. In addition, some covariates are also associated with MCI in older adults, such as gender, years of schooling, and starvation in childhood.

At the T1, T2, and T3 periods, the prevalence of MCI in Chinese older adults was 14.8%, 14.3%, and 16.9%, respectively, which was higher than the prevalence of MCI among the older adults in Zhejiang Province (13.0%) of China [55] and Taiyuan (9.7%) City [5] and lower than those in rural areas in northern China [6]. However, the prevalence of MCI in T4 was as high as 32.7%, which was far beyond the normal range of MCI prevalence among the older adults in China (estimated range: 9.7–23.3%) [8]. As older adults age, as does the risk of mild cognitive impairment. At T4, the average age of the older adults was 85.46 (SD = 8.21), of which 161 older adults reached or exceeded 100 years old, far exceeding the average life expectancy of China in 2021 of 78.2 years [56]. Therefore, among T4 respondents, the prevalence of MCI was as high as 32.7%, which is reasonable.

Numerous bioactive compounds and antioxidant nutrients (such as vitamins A, B, C, and E, carotenoids, flavonoids, and polyphenols) are naturally present in fruits, vegetables, and juices and are thought to reduce oxidative stress in the brain, thereby preventing age-related neurologic dysfunction [57,58,59]. Second, increasing fruit and vegetable intake has been associated with lower blood pressure and a lower risk of cardiovascular disease [60,61], both of which are established risk factors for MCI [62,63]. Existing research indicates that those who consume more folic acid age cognitively more slowly. Additionally, homocysteine builds up in brain tissue due to folic acid shortage, which may result in cognitive problems [64]. The development of cognitive decline, dementia, and AD in older people has been linked to elevated plasma total homocysteine, which has received widespread acceptance [65,66]. These results demonstrated the need for folic acid consumption to be considered in the daily prevention of cognitive deterioration. Fiber intake in vegetables and fruits may have a protective effect on cognitive function, and different sources of fiber intake, especially vegetable and fruit fiber, may be associated with different dimensions of cognitive function [67]. Consumption of dietary fiber can modulate the gut microbiota–brain system to influence cognitive and brain health. Additionally, the blood-pressure-lowering effects of dietary fiber intake may contribute to the preservation of healthy cognitive function among older adults [67].

An intriguing finding of this study is that frequent intake of either fruits or vegetables in isolation did not confer cognitive protection upon older adults, suggesting that the combined consumption of these two food groups elicits a synergistic effect exceeding that of their individual contributions—i.e., an effect akin to “1 + 1 > 2”. Notably, associations between multiple nutrients may potentiate the benefits of an individual nutrient [68]. Neuffer et al., for example, assessed plasma levels of LC n-3 PUFA, 25-hydroxyvitamin D, and carotenoids, indicating a fourfold increased risk of dementia among participants who demonstrated higher index values, indicating potential for mitigating cognitive decline through correction of multinutrient deficiencies [69]. In fact, the LipiDiDiet trial tested the efficacy of a multinutrient supplement in preventing Alzheimer’s disease progression, with follow-up results after 36 months, revealing significant long-term improvement in cognitive outcomes, brain atrophy, and disease progression among 81 participants (from an initial pool of 311 randomized subjects) [70].

Additionally, we discovered a positive correlation between older persons’ psychological well-being and their cognitive function. This agrees with the findings of further research [71,72]. In comparison to their cognitively similar-aged contemporaries, older individuals with high psychological well-being may have higher environmental mastery, a sense of purpose in life, and pleasant relationships with others [71]. However, it appears that depression and cognitive function is not always linked, which is consistent with the findings of previous studies [73]. Regardless of the fact that numerous studies have demonstrated that depression is linked to a higher likelihood of developing dementia [74,75] for reasons that are not clear. The simplest hypothesis is that they both share similar pathogenic pathways, with depression serving as a prodromal symptom of the diseases that ultimately lead to dementia [76]. In general, clinical–pathologic research has not indicated a link between depressed symptoms and diseases connected to dementia [77].

The relationship between some of the adjusted variables and cognitive function in this study is consistent with Longfei Jia’s study, such as age, sex, and years of schooling [8]. The aging process is known to impart alterations upon brain structures inclusive of systemic atrophy, as well as fragility of neurons present within memory-associated regions [78]. In females, rising prevalence rates of cognitive impairments may be attributed to reductions in estrogen and associated hormones following menopause, along with variations in brain morphology [79]. Notably, prolonged educational experiences may augment an individual’s cognitive reserve capacity [80]. Interestingly, this study failed to discern any potential association between birth location (rural vs. urban) or living alone with risk of developing MCI among older adults, thus diverging from established literature. It is plausible that gaps in the data obtained regarding respondents’ birthplaces might explain these unanticipated observations, whereby by some participants’ residences and birthplaces were distinct. Furthermore, results from this study indicate that living alone among aged individuals did not confer elevated risk for MCI. Notably, it is conceivable that this finding reflects the advanced age of the subjects evaluated, as few examinations have targeted populations, with a mean age exceeding 85.5 years (the average age of the older adults represented in the T4 timeframe).

The strengths of our study included the use of a 10-year follow-up and longitudinal analysis, which reduced the recollection bias and survival bias that frequently undermine the validity of the cross-sectional and retrospective study findings and the multiplex measures of eating patterns and cognitive function. Additionally, this sample is more typical of Chinese citizens since it includes 23 provinces or municipalities directly under the central government and is nationally representative. Furthermore, in order to reach a reliable estimate of the relevant connection, we carefully corrected for a few confounding factors. Most significantly, this study fills a research gap on older Asians by examining the association between fruit and vegetable consumption habits and MCI in older Chinese.

However, it is essential to be conscious of several research limitations. First, the selection bias and reduction in generality that may result from our inclusion and exclusion of individuals. Participants who did not completely engage in the T1–T4 survey were eliminated, as were those who passed away or were unreachable. Many sample sizes were reduced as a result of the older age during recruitment (mean age: 75.46 years). Second, as self-reported food frequency questionnaires are used in dietary surveys, there is a chance that fruit, vegetables, and their nutrient consumption will be misclassified. Additionally, for the frequency of intake of fruits and vegetables, only baseline data were included. As a result, we are unable to account for dietary modifications during the follow-up. The association discovered in this study may have been weakened as a result of these restrictions. Third, even though we have considered a few potential confounding variables, it is still challenging to completely exclude the impact of residual confounding variables (such as other nutrients, dietary practices, and health awareness) on the association between vegetable and fruit intake and MCI risk due to common risk factors. Finally, in cohort studies, when the incidence of outcomes is large, the OR value can be highly misleading because it exaggerates the size of the effect [81].

## 5. Conclusions

In younger Chinese older adults, MCI is estimated to afflict around 15% of the population, with prevalence rates increasing to 32.7% by 85 years of age; those aged around 90 years old reside in a particularly high-risk group. Of note, older adults who frequently consume both fruits and vegetables experience a reduction in MCI risk relative to those consuming these food groups infrequently—emphasizing the critical importance of regular intake of both fruits and vegetables in maintaining cognitive function. Encouragingly, there exists a positive correlation between psychological well-being (PWB) and cognitive function among older adults: individuals possessing robust PWB exhibited lower MCI risk profiles than their counterparts without such fortitude.

## Figures and Tables

**Table 1:** Overview and description of the sample.

Variable	T1		T2		T3		T4	
	MMSE = 0	MMSE = 1	MMSE = 0	MMSE = 1	MMSE = 0	MMSE = 1	MMSE = 0	MMSE = 1
	X ± S/N (%)		X ± S/N (%)		X ± S/N (%)		X ± S/N (%)	
Gender								
Men	1040 (49.7)	107 (29.6)	1038 (49.4)	107 (30.5)	2033 (50.7)	112 (27.0)	885 (53.6)	260 (32.4)
Women	1052 (50.3)	255 (70.4)	1065 (50.6)	244 (69.5)	1006 (49.3)	303 (73.0)	766 (46.4)	543 (67.6)
Age	74.29 ± 7.44	82.20 ± 9.19	77.55 ± 7.62	83.93 ± 9.44	80.35 ± 7.57	86.89 ± 9.06	83.25 ± 7.01	90.00 ± 8.63
Years of school	3.21 ± 3.80	1.02 ± 2.28	3.21 ± 3.80	0.96 ± 2.18	3.17 ± 3.76	1.32 ± 2.72	3.58 ± 3.90	1.47 ± 2.78
Ethnic								
Hans	1949 (93.2)	347 (95.9)	1974 (93.9)	322 (91.7)	1908 (93.6)	388 (93.5)	1538 (93.2)	758 (94.4)
Others	143 (6.8)	15 (4.1)	129 (6.1)	29 (8.3)	131 (6.4)	27 (6.5)	113 (6.8)	45 (5.6)
Birthplace								
Urban	184 (8.8)	19 (5.2)	185 (8.8)	18 (5.1)	176 (8.6)	27 (6.5)	152 (9.2)	51 (6.4)
Rural	1908 (91.2)	343 (94.8)	1918 (91.2)	333 (94.9)	1863 (91.4)	388 (93.5)	1499 (90.8)	752 (93.6)
Living condition								
Live with family	1784 (85.3)	277 (76.5)	1681 (79.9)	278 (79.2)	1595 (78.2)	306 (73.7)	1320 (80.0)	651 (81.1)
Live alone	296 (14.1)	79 (21.8)	400 (19.0)	67 (19.1)	423 (20.7)	102 (24.6)	303 (18.4)	126 (15.7)
Live in a nursing institution	12 (0.6)	6 (1.7)	22 (1.0)	6 (1.7)	21 (1.0)	7 (1.7)	28 (1.7)	26 (3.2)
Economic self-evaluation								
Good	306 (14.6)	86 (23.8)	269 (12.8)	99 (28.2)	172 (8.4)	81 (19.5)	141 (8.5)	100 (12.5)
Fair	1521 (72.3)	235 (64.9)	1426 (67.8)	222 (63.2)	1514 (74.3)	280 (67.5)	1106 (67.0)	599 (74.6)
Poor	274 (13.1)	41 (11.3)	408 (19.4)	30 (8.5)	353 (17.3)	54 (13.0)	404 (24.5)	104 (13.0)
Family annual income								
Q1	1614 (77.2)	275 (76.0)	1208 (57.4)	225 (64.1)	959 (47.0)	205 (49.4)	628 (38.0)	295 (36.7)
Q2	275 (13.1)	35 (9.7)	424 (20.2)	42 (12.0)	534 (26.2)	103 (24.8)	412 (25.0)	226 (28.1)
Q3	57 (2.7)	10 (2.8)	200 (9.5)	24 (6.8)	243 (11.9)	46 (11.1)	216 (13.1)	95 (11.8)
Q4	146 (7.0)	42 (11.6)	271 (12.9)	60 (17.1)	303 (14.9)	61 (14.7)	395 (23.9)	187 (23.3)
Marital status								
Currently married and living with spouse	1300 (62.1)	133 (36.7)	1090 (51.8)	120 (34.2)	1052 (51.6)	122 (29.4)	782 (47.4)	185 (23.0)
Currently married and not living with spouse	65 (3.1)	6 (1.7)	54 (2.6)	8 (2.3)	38 (1.9)	11 (2.7)	35 (2.1)	17 (2.1)
Unmarried or other	727 (34.8)	223 (61.6)	959 (45.6)	223 (63.5)	949 (46.5)	282 (68.0)	834 (50.5)	601 (74.8)
Timely treatment								
Yes	1973 (94.3)	318 (87.8)	2018 (96.0)	319 (90.9)	1999 (98.0)	386 (93.0)	1614 (97.8)	769 (95.8)
No	119 (5.7)	44 (12.2)	85 (4.0)	32 (9.1)	40 (2.0)	29 (7.0)	37 (2.2)	34 (4.2)
Starving in childhood								
Yes	1551 (74.1)	289 (79.8)	1553 (73.8)	287 (81.8)	1503 (73.7)	337 (81.2)	1206 (73.0)	634 (79.0)
No	541 (25.9)	73 (20.2)	550 (26.2)	64 (18.2)	536 (26.3)	78 (18.8)	445 (27.0)	169 (21.0)
Vegetable and fruit intake patterns								
V−/F−	120 (5.7)	56 (15.5)	99 (4.7)	35 (10.0)	117 (5.7)	61 (14.7)	105 (6.4)	137 (17.1)
V+/F−	1043 (49.9)	194 (53.6)	1105 (52.5)	238 (67.8)	1021 (50.1)	231 (55.7)	786 (47.6)	397 (47.2)
V−/F+	20 (1.0)	5 (1.4)	15 (0.7)	7 (2.0)	19 (0.9)	6 (1.4)	16 (1.0)	14 (1.7)
V+/F+	909 (43.5)	107 (29.6)	884 (42.0)	71 (20.2)	882 (43.3)	117 (28.2)	744 (45.1)	273 (34.0)
PWB	26.54 ± 3.65	26.64 ± 6.59	27.47 ± 4.06	26.06 ± 5.68	27.26 ± 4.37	27.00 ± 6.74	-	-
Depression								
No	-	-	1757 (83.5)	268 (76.4)	1758 (86.2)	349 (84.1)	-	-
Yes	-	-	346 (16.5)	83 (23.6)	281 (13.8)	66 (15.9)	-	-
All	2092 (85.2)	362 (14.8)	2103 (85.7)	351 (14.3)	2039 (83.1)	415 (16.9)	1651 (67.3)	803 (32.7)

Note: PWB denotes psychological well-being. S denotes Standard deviation. N denotes Number.

**Table 2:** Factors associated with MCI among Chinese older adults.

Factors Associated with Cognitive Function	Model 1 Unadjusted			Model 1 (T1–T4) Adjusted			Model 2 (T2–T3) Adjusted		
	OR	95% CI of OR		OR	95% CI of OR		OR	95% CI of OR	
		LB	UB		LB	UB		LB	UB
Gender = Female (ref: Male)	0.961 ***	0.95	0.973	0.962 ***	0.951	0.974	0.958 ***	0.944	0.971
Age	0.992 ***	0.991	0.993	0.992 ***	0.991	0.993	0.994 ***	0.992	0.995
Years of schooling	1.005 ***	1.004	1.007	1.005 ***	1.003	1.006	1.004 **	1.001	1.006
Ethnic = Others (ref: Hans)	0.998	0.974	1.022	1.000	0.977	1.025	1.012	0.984	1.04
Birthplace = Rural (ref: Urban)	0.981 *	0.963	0.999	0.982	0.964	1.001	0.989	0.965	1.014
Living condition = Live in a nursing institution (ref: Live with family)	0.984	0.872	1.109	0.983	0.873	1.106	0.978	0.898	1.065
Living condition = Live alone (ref: Live with family)	1.016	0.996	1.036	1.016	0.996	1.037	1.021 *	1.001	1.041
Economic self-evaluation = Good (ref: poor)	1.012	0.991	1.034	0.999	0.978	1.021	1.018	0.992	1.044
Economic self-evaluation = Fair (ref: poor)	1.006	0.989	1.023	0.999	0.982	1.016	1.004	0.983	1.026
Family income = Q4 (ref: Q1)	1.009	0.988	1.03	1.005	0.984	1.026	1.003	0.983	1.023
Family income = Q3 (ref: Q1)	0.999	0.961	1.038	0.998	0.96	1.037	0.982	0.957	1.007
Family income = Q2 (ref: Q1)	1.013	0.995	1.03	1.009	0.992	1.026	0.994	0.978	1.01
Marital status = Unmarried or other (ref: currently married and living with spouse)	0.983 *	0.969	0.998	0.986	0.971	1.001	0.987	0.972	1.002
Marital status = Currently married and not living with spouse (ref: currently married and living with spouse)	0.996	0.968	1.025	1.000	0.971	1.029	0.958	0.911	1.006
Timely treatment = No (ref: Yes)	0.978	0.952	1.004	0.985	0.959	1.012	0.982	0.943	1.023
Starving in childhood = No (ref: Yes)	1.019 **	1.008	1.031	1.019 **	1.008	1.03	1.014 *	1.001	1.027
Vegetable and fruit intake patterns = V+/F+ (ref: V−/F−)	1.05 **	1.021	1.08	1.026 *	1.001	1.053	1.101 ***	1.062	1.141
Vegetable and fruit intake patterns = V−/F+ (ref: V−/F−)	1.038	0.978	1.101	1.028	0.964	1.097	0.995	0.869	1.14
Vegetable and fruit intake patterns = V+/F− (ref: V−/F−)	1.036 *	1.008	1.065	1.014	0.989	1.04	1.075 ***	1.038	1.114
PWB	1.008 ***	1.006	1.01	1.003 **	1.001	1.005	1.002 *	1	1.004
Depression = Yes (ref: No)	-	-	-	-	-	-	0.985	0.967	1.003

Note: OR denotes odds ratio, CI denotes confidence interval, LB denotes lower bound, UB denotes upper bound. * *p* < 0.05; ** *p* < 0.01; *** *p* < 0.001. PWB denotes psychological well-being.

**Table 3:** Factors associated with MCI among Chinese older adults (Model 3).

Variable	*p*	OR	95% CI of OR	
			LB	UB
[F = 1] ref [F = 0]	0.189	0.989	0.972	1.006
[V = 1] ref [V = 0]	0.348	1.006	0.994	1.018

Note: F = 1 denotes frequent fruit consumption, F = 0 denotes infrequent fruit consumption, V = 1 denotes frequent vegetables consumption, V = 0 denotes infrequent vegetables consumption, OR denotes odds ratio, CI denotes confidence interval, LB denotes lower bound, UB denotes upper bound, controlling for gender, years of schooling, ethnic, birthplace, living condition, economic self-evaluation, family income, marital status, timely treatment, and starving in childhood.

## Data Availability

The raw data supporting the conclusions of this article can be found here: https://opendata.pku.edu.cn/dataverse/CHADS (accessed on 20 December 2022).
